# Peripheral T-cell lymphoma of the lip: a rare case unveiling key insights into diagnosis and management

**DOI:** 10.3332/ecancer.2025.1998

**Published:** 2025-09-25

**Authors:** Tooba Ali, Laraib Khan, Bilal Mazhar Qureshi, Asim Hafiz, Maria Tariq, Khurram Minhas, Nasir Ali, Ahmed Nadeem Abbasi

**Affiliations:** 1 Department of Oncology, Section Radiation Oncology, Aga Khan University Hospital, Karachi 74800, Pakistan; 2 Department of Histopathology, Aga Khan University Hospital, Karachi 74800, Pakistan

**Keywords:** primary T-cell lymphoma P-TCL, radiotherapy, diagnostic challenge, extra nodal lymphoma, rare subsites, lip, multidisciplinary management

## Abstract

Peripheral T-cell lymphomas (PTCLs) represent a rare and heterogeneous group of lymphoproliferative disorders, accounting for about 10% of non-Hodgkin lymphomas. While PTCLs typically present at nodal sites, extra nodal involvement is uncommon, particularly in the oral cavity. This case report presents a rare instance of peripheral T-cell lymphoma, not otherwise specified (PTCL-NOS), manifesting as a persistent lesion on the lower lip in a 70-year-old male patient. The patient underwent multiple biopsies, which required immunohistochemical staining to confirm the diagnosis. Initial histopathological examinations raised suspicion of a lymphoproliferative disorder, with further testing revealing a 4.5 × 1.5 × 2.8 cm Fluorodeoxyglucose (FDG)-avid lesion on positron emission tomography (PET)/CT. The lesion was confirmed to be PTCL-NOS, characterised by positive CD3 and CD56 markers and a high Ki-67 proliferative index. Treatment involved six cycles of CHOEP chemotherapy followed by consolidative radiation therapy, delivering a total dose of 36 Gy. The patient responded well to treatment, with an interim PET scan showing a complete metabolic response (Deauville score of 3). Follow-up visits confirmed the absence of residual or recurrent disease. A teleconsultation a 6-month post-radiotherapy, along with an examination by a plastic surgeon, also showed no signs of recurrence. This case highlights the diagnostic challenges associated with PTCL at rare non-nodal sites and underscores the importance of a multidisciplinary approach in managing such cases. The patient remains in remission, with ongoing surveillance recommended for up to 5 years to monitor for potential disease recurrence. Further studies and long-term follow-up of similar cases are warranted to better understand the behaviour and optimal treatment strategies for PTCLs in rare extra nodal locations.

## Background

Lymphomas, categorised by the World Health Organisation (WHO) classification into Hodgkin and non-Hodgkin lymphoma (NHL), represent a diverse group of malignancies that originate from lymphoepithelial cells [1]. NHL constitutes 86% of all lymphomas and is the second most common type of head and neck malignancy after squamous cell carcinoma. Extra nodal lymphomas account for 25%–40% of all NHL cases [2]. Based on the Revised European American Classification of Lymphoid Neoplasms, lymphomas are currently classified according to the WHO classification, which recognises over 20 different subtypes of NHL and considers their morphology, immunophenotype, genetic characteristics and clinical features, while stratifying it. Oral cavity lymphomas make up to 3.5% of all lymphomas with less commonly affected subsites including lips and uvula, as backed up by literature [3]. In our region, the diagnosis and management of peripheral T-cell lymphoma (P-TCL) is supported only by case reports and literature review, with less than 2% of P-TCL cases involving oral cavity subsites [2, 4]. The pathogenesis of this entity revolves around improper maturation of the T-cell lineage, often occurring in the context of immunodeficiency states or viral infections such as AIDS, autoimmune disorders and viruses like Human Herpes Virus and Epstein-Barr virus, as documented in a limited number of case reports in the literature [5].

Among NHL in the head and neck region, Burkitt’s lymphoma is the most common type, affecting the maxilla, mandible and premolar or molar areas, particularly in paediatric patients. It is endemic to the African region, with a key histopathological feature being the ‘starry sky’ appearance due to non-cleaved B-cell lymphocytes. Immunohistochemical (IHC) staining for CD20, CD43 and CD79 assisting in the diagnosis [6]. In diagnosing P-TCL of lip histopathological assessment of specimen and IHC staining was of prime importance. CD3 serves as a pan T-cell marker and is found on the majority of mature T and NK-cell lymphomas, except in cases of anaplastic large cell lymphoma (ALCL). Further IHC staining helps differentiate other T-Cell lymphomas from peripheral T-cell lymphoma, not otherwise specified (P-TCL NOS); these include CD4, CD5, CD8 and CD20 [7,8].

## Case presentation

**Figure 1.**
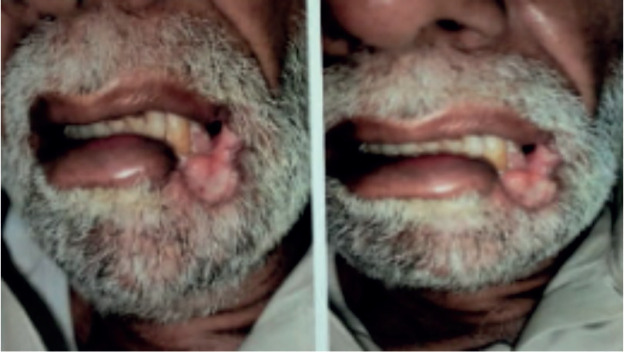
Left lower lip lesion at initial presentation.

An elderly gentleman in his early 70s, with no significant past medical history or prior comorbidities, and from a low socioeconomic background, was referred to the radiation oncology clinic for a persistent left-sided lower lip lesion that had lasted for 5 months (Figure 1). An initial biopsy of the left lower lip lesion did not indicate invasive squamous cell carcinoma but raised suspicion of a lymphoproliferative disorder. Seeking a second opinion, a histopathological examination confirmed the presence of squamous mucosa with ulceration and necro-inflammatory debris. The primary physician subsequently recommended a contrast-enhanced CT scan of the head and neck, which revealed a 4.1 × 1.7 cm ulcerated lesion on the left buccal mucosa, closely abutting the body of the left hemimandible medially, along with multiple non-enhancing sub-centimetric neck nodes.

An incisional biopsy of the left lower lip was performed, which showed benign squamous epithelium alongside atypical lymphoid cells of concern. Given these findings, a lymph node biopsy was recommended. Further investigations, including a contrast-enhanced CT scan of the neck, chest, abdomen and pelvis, indicated interval progression of the left lower lip lesion and prominent bilateral axillary, portocaval, abdominal, iliac and inguinal lymph nodes, raising suspicion for a possible lymphoproliferative disorder. A biopsy of the left axillary lymph node, accompanied by IHC stains for CD20, CD3 and Ki-67 (Mib-1), displayed a reactive pattern, effectively ruling out granuloma or malignancy.

**Figure 2.**
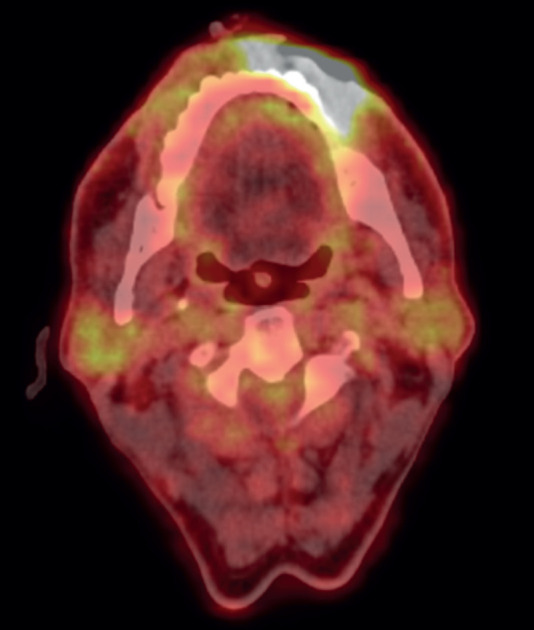
Pre-chemotherapy PET CT scan, showing FDG avid lesion involving left lower lip.

**Figure 3.**
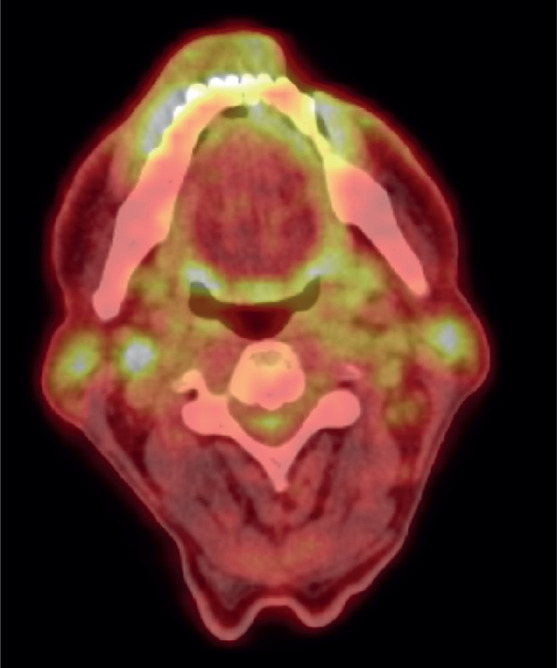
Post-chemotherapy PET CT scan, showing FDG avid residual left lower lip lesion.

To assess the extent of the condition, an 18F-fluorodeoxyglucose positron emission tomography (PET)/CT scan was performed, revealing an Fluorodeoxyglucose (FDG)-avid left lower lip lesion measuring 4.5 × 1.5 × 2.8 cm with a maximum standardised uptake value (max) of 15.66 (Figure 2). A repeat biopsy and IHC profiling of the left lower lip lesion demonstrated positivity for CD3 and CD56, a loss of CD5 staining in CD3-positive cells, a reserved CD4/CD8 ratio and negativity for CD20, TdT and EBV status. Additionally, a Ki-67 proliferative index of approximately 50% further supported the diagnosis of P-TCL NOS (Figure 3).

Although P-TCL is commonly associated with an immunosuppressive state, this patient had no underlying immunosuppressive conditions, making this case an atypical presentation.

### Treatment

**Figure 4.**
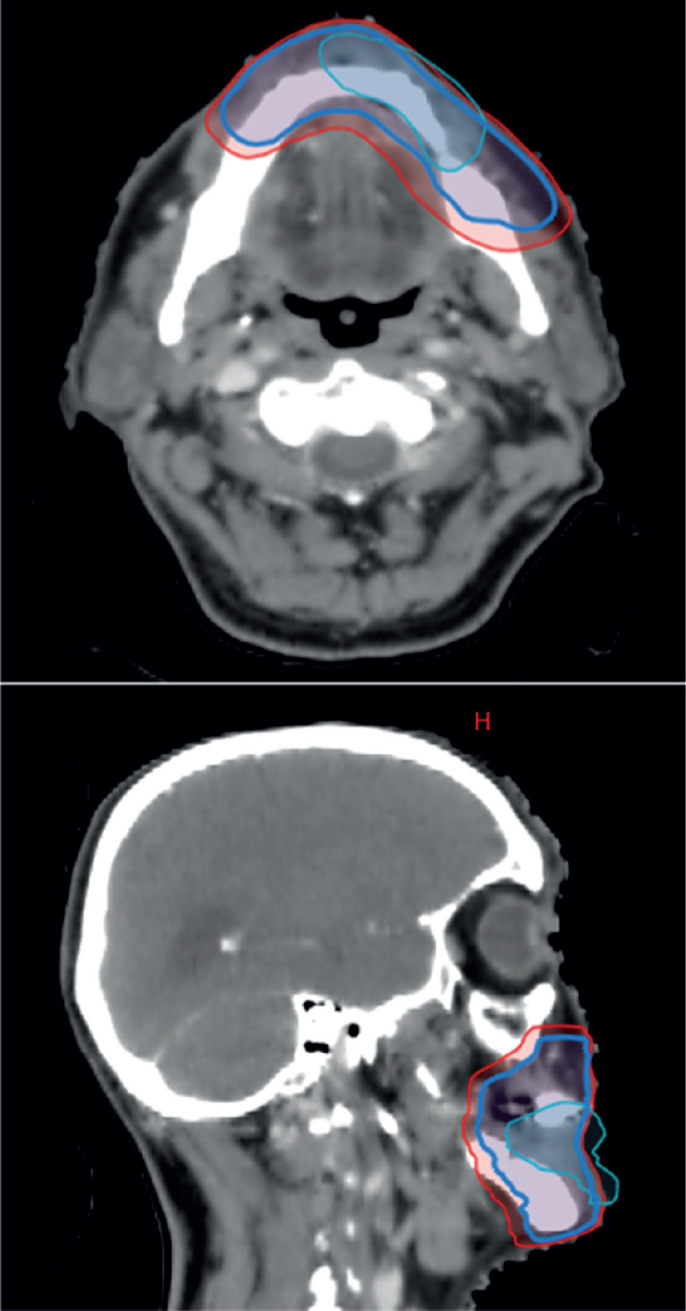
CT-based RT planning, representing pre-chemotherapy GTV, which has now regressed in cyan, CTV and PTV in red.

After a thorough discussion in the multidisciplinary lymphoma tumour board, the patient was started on a chemotherapy regimen consisting of Cyclophosphamide 750 mg/m² IV on day 1, Doxorubicin 50 mg/m² IV on day 1, Vincristine 1.4 mg/m² (max 2 mg) IV on day 1, Etoposide 100 mg/m² IV on days 1–3 and Prednisone 100 mg PO on days 1–5, administered over 6 cycles every 3 weeks. This regimen was well-tolerated by the patient. Interim PET scan was repeated after 4 cycles of chemotherapy, which showed complete metabolic response (Deauville 3). After the completion of six cycles, an end-of-treatment PET-CT was done, which revealed complete metabolic response (Deauville 3) with mild FDG-avidity over lip lesion and complete resolution of the FDG-avid lymph nodes (Figure 4).

**Figure 5.**
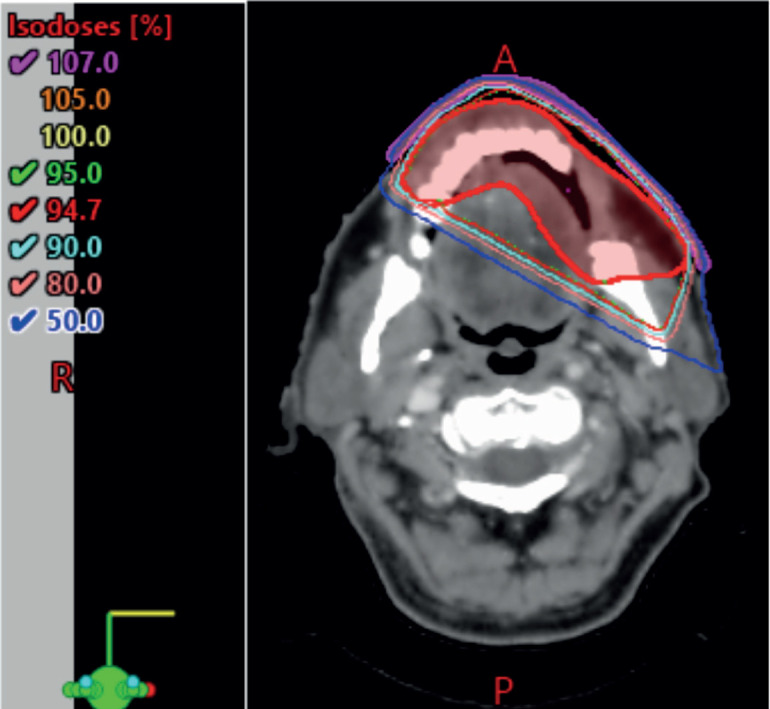
3DCRT plan showing isodose curves.

**Figure 6.**
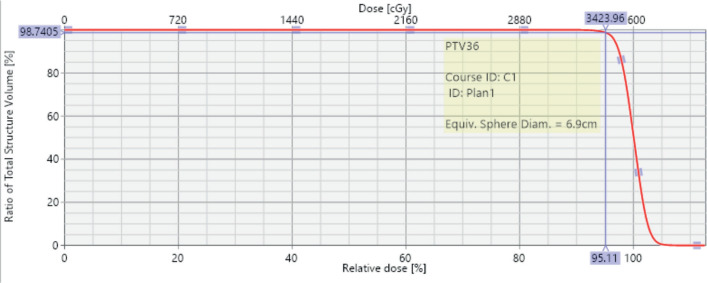
Dose volume histogram showing PTV coverage with 95% of the volume being covered with 95% isodose line.

**Figure 7.**
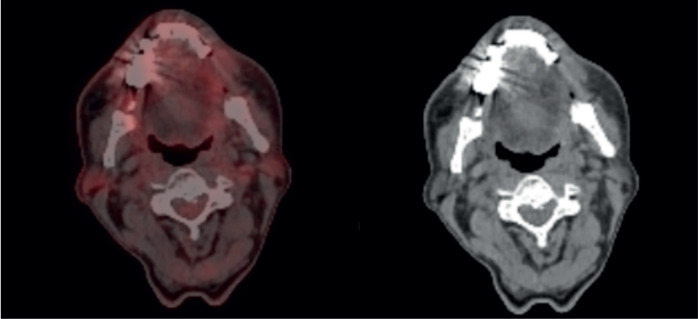
Post treatment follow-up PET CT scan, showing complete metabolic response.

The case was revisited in the tumour board meeting, where the consensus was to proceed with consolidative radiotherapy (RT). Within 6 weeks of completing the sixth cycle of chemotherapy, the patient was scheduled for 3D conformal radiation therapy (3DCRT). Treatment planning done was involved site radiotherapy (ISRT) in which previously Pet avid site of primary lesion was contoured as pre chemo gross tumour volume (GTV) then a clinical target volume (CTV) was generated to include entire site (ISRT) in our radiation field followed by planning target volume (PTV) to incorporate set up error changes. A total dose of 3,600 cGy was delivered in 18 fractions, with 200 cGy per fraction, using 6 MeV energy and a 3 mm bolus to ensure adequate surface dose distribution (Figures 5–7).

During RT, the patient was monitored weekly. By the second week, he developed grade 2 dermatitis and mucositis, which were managed conservatively with topical steroid ointments and mouthwashes containing pain killers and anti-inflammatory agents. This weekly review approach ensured that the patient could tolerate and complete the RT without any interruption, aligning with what has been observed in other cases where comprehensive planning and review, followed by complete radiation delivery radio biologically improves local control [9].

### Outcome and follow-up

**Figure 8.**
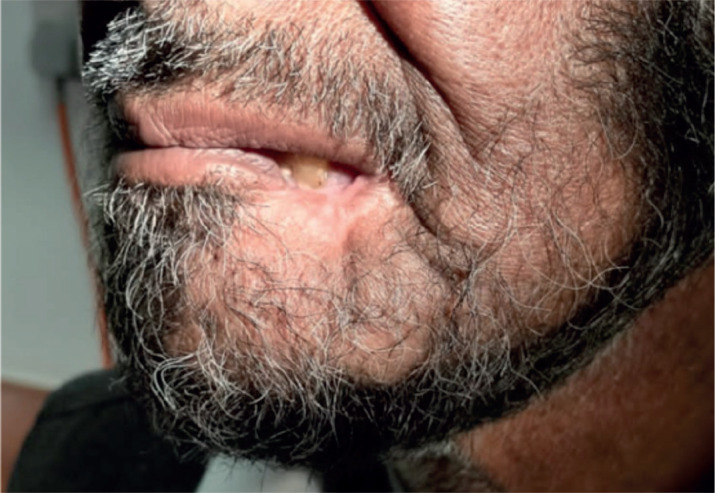
Complete resolution of left lower lip lesion on after treatment on follow up.

During the 1-month follow-up visit after RT, the patient’s dermatitis and mucositis had resolved. He was advised to return for a follow-up appointment in 2 months, at which time a PET-CT scan was performed. This scan demonstrated a sustained complete metabolic response (Figure 8), and clinical examination revealed no evidence of residual or recurrent disease.

Following these encouraging results, the patient was referred to a plastic surgeon for surgical reconstruction of the lip. During a teleconsultation at 6 months post- RT, a local examination conducted by the plastic surgeon revealed no evidence of disease recurrence. However, the patient was advised to continue annual surveillance with evaluation by a primary surgeon along with PET/CT scans, as clinically indicated, for up to 5 years following the completion of treatment.

## Discussion

Extra nodal involvement in Hodgkin lymphoma is rare, occurring in approximately 1% of cases, compared to 23%–30% in NHL. The Waldeyer’s ring is the most common site for NHL (36%), while NHL in the oral cavity is relatively uncommon, accounting for only 2% of cases. The hard palate and alveolus are the most frequently affected subsites, with other reported locations including the oral commissure, tongue, uvula, gingiva-buccal sulcus, palate and maxilla [10].

In a case series by Shah *et al* [10], the typical clinical presentation was painless, progressive swelling, without B symptoms such as fever, weight loss or night sweats. Among the 15 cases discussed, the alveolus and hard palate were the most common sites of presentation (*n* = 5 each). Only two patients had established immunosuppressive conditions (HIV). Most patients received a combination of chemotherapy and radiation therapy, with surgery reserved for biopsy in 10 out of 15 cases. Follow-up data were available for 11 out of 15 patients after 2 years of treatment with neoadjuvant chemotherapy and consolidative radiation therapy. Of these, five patients showed no evidence of local or distant disease [11].

Kaplan *et al* [11], in a recent literature review of 23 cases (7 new and 16 from the literature), found that the lower lip was the most affected site (16 cases, 69.56%). Fourteen cases (60.87%) were limited to the lips, while 8 (34.78%) were multifocal. Nine cases (39.13%) were associated with Sjögren’s syndrome, with one patient also having Hashimoto’s thyroiditis. IgG4-related disease and HIV were each reported in one case. In most cases (19, 82.6%), the mucosal lip or salivary glands were involved, with only three cases (13.6%) showing cutaneous involvement. The typical presentation was single or multiple nodules (15 cases, 65.21%), with surface ulceration seen in only two cases (8.69%). No constitutional symptoms were reported, though paraesthesia was noted in one case (4.34%). Most cases (18, 78.26%) were extra nodal marginal zone B-cell lymphoma (MALT lymphoma), with one case each of mantle cell lymphoma, NK-T cell lymphoma, CD30-positive lymphoma and plasmablastic lymphoma. Notably, only one case of P-TCL was reported in this review [11].

PTCLs are a diverse group of lymphoproliferative disorders derived from mature T cells, representing approximately 10% of NHLs. The most common subtype is PTCL-NOS; 26%, followed by angioimmunoblastic T-cell lymphoma (19%), ALCL with anaplastic lymphoma kinase (ALK)-positive (7%) and ALK-negative (6%) forms, and enteropathy-associated T-cell lymphoma (<5%). However, P-TCL typically presents at nodal sites, our case involved a presentation at one of the rarest non-nodal subsites [12].

PTCL NOS, includes all PTCLs that do not fit into other specific categories. PTCL NOS tumour cells are typically CD3-positive, and CD3 staining is more effective than H&E staining in highlighting cytologic atypia in lymphocytes. It is critical to assess for cytologic atypia when reviewing CD3-stained slides, and institutions should ensure quality control in immunostaining techniques, tumour cells generally express either CD4 or CD8, although they may sometimes lack both markers. While both CD4- and CD8-positive cells may appear together under low magnification, it is rare for a single cell to express both markers, requiring careful interpretation. CD56 expression can vary across PTCL subtypes, and β-F1 and T-cell receptor delta staining can help distinguish between αβ and γδ T cells. CD30 staining is increasingly used in T-cell lymphomas, particularly with the development of new treatments targeting CD30-positive tumour cells [7].

Anthracycline-based chemotherapy regimens, such as cyclophosphamide, doxorubicin, vincristine and prednisone (CHOP), CHOP plus etoposide (CHOEP) or dose-adjusted etoposide, prednisone, vincristine, cyclophosphamide and doxorubicin, are the most used first-line treatments, as they have shown a tendency toward reducing mortality. In a retrospective analysis of 289 patients with PTCL treated in the DSHNHL trials, CHOEP was found to be associated with improved event-free survival [13].

In a recent case report, Bianco *et al* [14] emphasised the important role of radiation therapy in treating localised NHL of the lip that was unresponsive to induction chemotherapy. The patient achieved a complete response after receiving conventional radiation therapy (4 Gy in 2 fractions via 3DCRT with bolus placement), and this response was sustained for 1 year during follow-up. According to the NHL Classification Project, PTCL accounts for 8%–10% of all NHLs, with clinical presentations ranging from indolent to aggressive and potentially fatal [14].

In the era of PET scan imaging for lymphomas response criteria known as the Lugano criteria has been established since 2014 for assessing response using PET/CT scans, following the 5-point (PS) system. The 5-PS relies on the visual evaluation of FDG uptake in the affected areas compared to that of the mediastinum and liver. A score of 1 indicates no abnormal FDG avidity, while a score of 2 signifies uptake less than that of the mediastinum. A score of 3 represents uptake greater than the mediastinum but less than the liver, whereas scores of 4 and 5 indicate uptake greater than the liver, and greater than the liver with new sites of disease, respectively. With our patient showing Deauville 3 response after completion of chemotherapy [15].

As per the NHL Classification Project, PTCL constitutes a total of 8%–10% of all non-Hodgkin with varied clinical patterns ranging from indolent to aggressive to potentially fatal [10].

Additionally, certain sites have demonstrated a poor response to chemotherapy and radiation therapy, as observed in cases of uvular and nasal PTCL [11, 16]. For example, a case of PTCL of the uvula in a 54-year-old patient initially showed disease remission after three cycles of CHOP chemotherapy but experienced disease progression and ultimately succumbed after the fourth cycle of treatment [17]. Another rare manifestation seen in the case of Nasal T Cell Lymphoma, despite the absence of systemic disease, a delay in the diagnosis occurred due to atypical disease manifestation with aggressive disease showing poor response to chemotherapy [18].

Literature on complete remission in rare sites of PTCL is limited; however, case reports support a complete response in PTCL of the oral commissure, with disease remission and no local recurrence after a 3-year follow-up when treated initially with RT alone [19]. Similarly, in our case, the patient's disease, located at the lip commissure, was treated with six cycles of R-CHOP chemotherapy followed by consolidative radiation therapy. A PET scan performed 3 months post-treatment completion showed a complete metabolic response.

Evidence supporting the role of radiation in T-cell lymphoma of the lip underscores the importance of maintaining quality with daily peer reviews of radiation therapy treatment contours and plans. Qureshi *et al* [20] emphasised the value of daily peer review meetings before commencing radiation therapy, which resulted in 12.9% minor adjustments and 8.6% major changes to treatment plans. Notably, more changes were made in radical treatment contours compared to those for palliative patients (92.3% versus 7.7%) [20]. Overall, for patients with early-stage PTCL, the role of consolidative radiation therapy following combination chemotherapy remains debated. Retrospective studies offer conflicting evidence regarding its benefit, with some suggesting improved local control and survival, while others find no significant advantage. This reflects the variability in treatment outcomes and the need for individualised approaches based on disease characteristics and patient factors [21, 22].

## Conclusion

In conclusion, this case report poses a rare presentation of PTCL holding a diagnostic challenge warranting multiple biopsies and IHC staining followed by extensive systemic treatment with consolidative radiation therapy, implying the need to extensively study such cases with long-term follow-up required to understand disease behaviour and adequate treatment options in a multidisciplinary setting.

## Conflicts of interest

The author(s) declare that they have no conflicts of interest.

## Funding

None.

## Informed consent

Written consent for publication was obtained from the patient.

## Author contributions

Tooba Ali: Manuscript writing, Laraib Khan: Drafting, Bilal Mazhar Qureshi: Review, Asim Hafiz: Review, Maria Tariq: Review, Khurram Minhas: Pathology Imaging and Manuscript Review, Nasir Ali: Proof reading and Ahmed Nadeem Abbasi: Conceptualisation.
